# Change of Scaling-Induced Proinflammatory Cytokine on the Clinical Efficacy of Periodontitis Treatment

**DOI:** 10.1155/2015/289647

**Published:** 2015-03-25

**Authors:** Kou-Gi Shyu, Cheuk-Sing Choy, Daniel Chung-Lang Wang, Wei-Chen Huang, Shyuan-Yow Chen, Chien-Hsun Chen, Che-Tong Lin, Chao-Chien Chang, Yung-Kai Huang

**Affiliations:** ^1^Division of Cardiology, Shin Kong Wu Ho-Su Memorial Hospital, Taipei 111, Taiwan; ^2^Graduate Institute of Clinical Medicine, Taipei Medical University, Taipei 110, Taiwan; ^3^Department of Medicine, School of Medicine, College of Medicine, Taipei Medical University, Taipei 110, Taiwan; ^4^Department of Emergency, Min-Sheng General Hospital, Taoyuan 330, Taiwan; ^5^Department of Obstetrics and Gynecology, Min-Sheng General Hospital, Taoyuan 330, Taiwan; ^6^Dental Department, Cathay General Hospital, Taipei 106, Taiwan; ^7^School of Dentistry, College of Oral Medicine, Taipei Medical University, Taipei 110, Taiwan; ^8^Department of Cardiology, Cathay General Hospital, Taipei 106, Taiwan; ^9^School of Oral Hygiene, College of Oral Medicine, Taipei Medical University, Taipei 110, Taiwan

## Abstract

Proinflammatory cytokines are key inflammatory mediators in periodontitis. This study aimed to investigate the relationship between proinflammatory cytokines in saliva and periodontal status. To investigate the usefulness of cytokines in the therapeutic approach for periodontal disease, the relationship between stimulated cytokine changes and the periodontitis treatment outcome was investigated in this study. Saliva was obtained from 22 patients diagnosed by dentists as having chronic periodontitis. The proinflammatory cytokine (interleukin-1*α* (IL-1*α*), interleukin-1*β* (IL-1*β*), interleukin-6 (IL-6), interleukin-8 (IL-8), tumor necrosis factor *α* (TNF-*α*), and tumor necrosis factor *β* (TNF-*β*)) levels were determined using a commercially available kit. The IL-1*β* and IL-6 levels increased, whereas the TNF-*β* levels decreased with the severity of periodontitis (4 mm pocket percentage). Poststimulation IL-1*α*, IL-6, and IL-8 levels were higher in patients who had an improved treatment outcome. The differences of IL-6 levels (cut point: 0.05 *μ*g/g) yielded a sensitivity and specificity of 90.0% and 81.82%, respectively, for predicting the periodontitis treatment outcome. Among the proinflammatory cytokines, stimulated IL-6 was an excellent marker for predicting the periodontitis treatment outcome.

## 1. Introduction

Periodontal disease is a localized inflammatory disorder in which periodontal pathogens escape the host immunological defense system, leading to tissue destruction and bone loss [1, 2]. Current knowledge suggests that periodontal pathogenesis is a mixed host response to dental bacterial biofilms and proinflammatory mediators [3]. A balance of these proinflammatory mediators and the host immune response can determine the effectiveness of a clinical treatment for periodontal disease.

These proinflammatory mediators are activated when bacterial biofilms accumulate in the gingival area of the teeth. In vitro studies have shown that proinflammatory cytokines (interleukin-1*α* (IL-1*α*), interleukin-1*β* (IL-1*β*), interleukin-6 (IL-6), interleukin-8 (IL-8), tumor necrosis factor *α* (TNF-*α*), and tumor necrosis factor *β* (TNF-*β*)) production increased when human gingival fibroblasts were stimulated by* Porphyromonas gingivalis* (*P. gingivalis*) [4], which also stimulated periodontal ligament stem cells to produce IL-1*β*, IL-6, and IL-8 [5]. These proinflammatory cytokines can both potentially influence the progression of periodontal disease and be a novel therapeutic target for chronic periodontitis treatment.

Periodontitis is a type of inflammatory disease with risk factors including periodontal pathogens, the lifestyle, psychosocial factors, chronic diseases, and genetic factors [6]. Recent epidemiological studies have reported that proinflammatory cytokines are associated with periodontitis [7, 8]. Salivary IL-1*β* levels increase with the severity of periodontitis [9]. Interleukin-6 correlates with the presence of periodontitis; however, the TNF-*α* levels do not differ between periodontitis cases and healthy subjects [10]. Although proinflammatory cytokines are related to periodontitis, no study has yet investigated whether these cytokines interact or influence periodontitis outcomes.

Osteoclastogenesis is a key process leading to clinical periodontitis outcomes. Proinflammatory cytokines (IL-1*α*, IL-1*β*, IL-6, IL-8, TNF-*α*, and TNF-*β*) are associated with osteoclastogenesis [11]. These proinflammatory cytokines decreasing had reflected the clinical efficacy of periodontitis treatment [12]. The levels of these proinflammatory cytokines decrease when patients show clinical improvements during periodontal treatment [10].

Scaling is a part of periodontal disease treatment. Scaling, involving the thorough mechanical debridement of dental calculus, not only is a treatment for periodontal disease but also stimulates gingival tissue. Studies have shown that low intensity pulsed ultrasound activates the cell growth signaling pathway and stimulates human circulating angiogenic cells, which release endothelial nitric oxide synthase [13, 14]. The proinflammatory cytokine expression in gingival tissues increases with the severity of inflammation [15]. This study hypothesized that a scaling-stimulated change in the proinflammatory cytokine profile is related to periodontitis treatment effectiveness. The study aimed to investigate the relationship between salivary proinflammatory cytokines and periodontal status. The relationship between scaling-stimulated changes in proinflammatory cytokines and periodontal treatment effectiveness is evaluated in this study.

## 2. Material and Methods

### 2.1. Saliva and Clinical Data Collection

Saliva was collected from 22 systemically healthy patients with chronic periodontitis (at least 6 pockets with pocket depths (PDs) of >5 mm and more than 16 functional teeth) over a 6-month period (October 2011 to March 2012) at Cathay General Hospital Dental Department. Before conducting interviews and collecting samples, written informed consent was obtained from all subjects. Structured questionnaires were distributed by an experienced assistant who obtained data related to socioeconomic, demographic, and lifestyle characteristics through personal interviews. The study complied with the World Medical Association* Declaration of Helsinki* and was approved by the Cathay General Hospital Institutional Review Board.

Periodontal diagnostic criteria were based on the classification of the American Academy of Periodontology [16]. Each patient received periodontal examination and treatment from the same dental clinician. The PD was evaluated as the distance between the gingival margin and the bottom of the sulcus/pocket and was assessed at 6 sites. Salivary samples were collected before and after patients received scaling. Subsequently, the patients completed the nonsurgical periodontal treatment procedure. Patients were regarded as part of the nonprogress (NP) treatment group when differences in >7 mm PD percentage increased between the initial clinical treatment and after the completion of 4 weeks of clinical treatment (*N* = 12). The remaining 10 patients were regarded as part of the effective treatment (ET) group (*N* = 10).

### 2.2. Saliva Preparation and Proinflammatory Cytokine Determination

We described the details of saliva collection in a previous paper [17]. In brief, saliva was collected using sterilized gauze pieces from the buccal and sublingual areas and recovered through centrifugation (1000 rpm, 3 min). At least 2 mL of unstimulated saliva was collected in each tube. The tubes were stored at −20°C and analyzed within 2 months. Proinflammatory cytokine (IL-1*α*, IL-1*β*, IL-6, IL-8, TNF-*α*, and TNF-*β*) levels were determined using a Human 22-plex multicytokine detection system (catalog number 48-011; Millipore, Billerica, MA, USA) and analyzed using a Luminex 100 system (Luminex Inc.). To consider changes in the salivary volume, we used the total protein level for adjustment while expressing the proinflammatory cytokines. The total protein level was evaluated using the Bradford test and spectrophotometric methods. The differences in proinflammatory cytokines were calculated by subtracting baseline salivary cytokine levels from scaling-stimulated salivary cytokines levels.

### 2.3. Statistical Analysis

Data were analyzed using SAS 9.3 software (SAS, Cary, NC, USA). Because the salivary proinflammatory cytokines in this study were not normally distributed, a nonparametric test was used for the data analysis. Demographic characteristics and baseline clinical data for the NP and ET groups were analyzed using Fisher's exact test. Differences in continuous parameters between the NP and ET groups were analyzed using the Mann-Whitney *U* test. The correlation strength of proinflammatory cytokines before and after the scaling stimulation was determined using Spearman's rank correlation. To evaluate the accuracy of detecting periodontitis treatment effectiveness, receiver operating characteristic (ROC) curves and areas under the curve (AUC), based on the levels of these proinflammatory cytokines, were calculated. Probability levels of <0.05 were considered significant.

## 3. Results

Baseline clinical parameters, demographics, and conventional periodontitis risk factors of the treatment outcome group are shown in Table 1. No statistical differences existed in demographic characteristics or conventional periodontitis risk factors between the NP and ET groups.  Table 2 shows a comparison of the baseline cytokine levels between the NP and ET groups. No statistical differences existed in the baseline cytokine levels between the 2 groups.  Table 3 shows the correlations between baseline salivary proinflammatory cytokines in patients with periodontitis. Baseline salivary IL-1*β* was significantly correlated with baseline IL-1*α*, IL-6, IL-8, and TNF-*β*; the correlation coefficients were 0.46, 0.72, 0.60, and −0.46, respectively.

**Figure 1:**
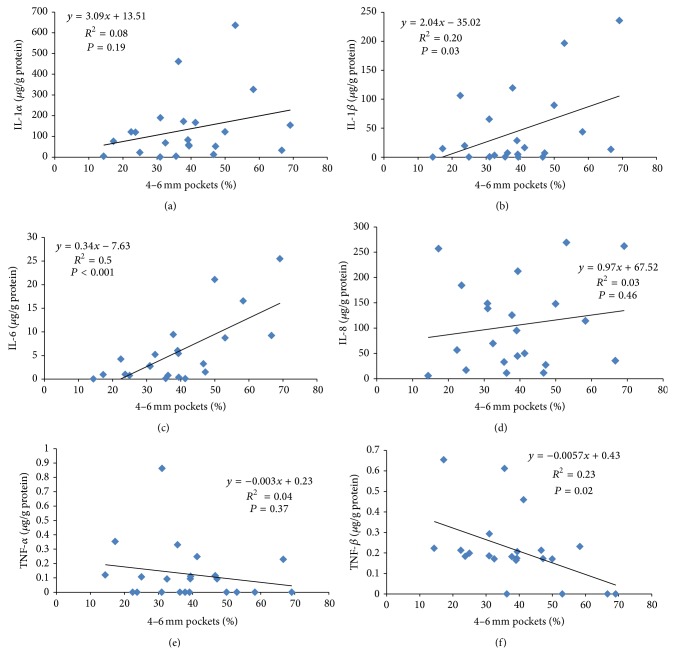
Scatter plots of baseline proinflammatory cytokines and 4–6 mm pocket percentages (before treatment) in patients with chronic periodontitis. (a) Interleukin-1*α* (IL-1*α*). (b) Interleukin-1*β* (IL-1*β*). (c) Interleukin-6 (IL-6). (d) Interleukin-8 (IL-8). (e) Tumor necrosis factor *α* (TNF-*α*). (f) Tumor necrosis factor *β* (TNF-*β*).

The scatter plots in Figure 1 show that proinflammatory cytokines and 4–6 mm pocket percentages are representative of the severity of periodontitis. The *β* values of IL-1*β* and IL-6 were 2.04 and 0.34, respectively, indicating that these 2 proinflammatory cytokines significantly increased with the severity of periodontitis. TNF-*β* significantly decreased with the baseline percentages of 4–6 mm pockets (*β* value = −0.0057 and *P* = 0.02).

To investigate the correlation between baseline and scaling-stimulated proinflammatory cytokines, the correlation coefficients between baseline and stimulated salivary cytokines in patients with periodontitis were calculated (Table 4). A positive correlation existed between baseline IL-1*α* and stimulated IL-1*α* (*r* = 0.66 and *P* < 0.01). The correlation between baseline IL-1*β* and stimulated IL-1*β* was also positive (*r* = 0.44 and *P* = 0.0.4). Stimulated IL-6 was significantly correlated with baseline IL-1*α*, IL-1*β*, IL-6, and TNF-*α*; the correlation coefficients were 0.63, 0.47, 0.60, and −0.48, respectively.

The relationship between proinflammatory cytokine differences and the clinical treatment outcome is shown in Table 5. The differences in IL-1*α*, IL-6, and IL-8 were significantly higher in the ET group than in the NP group. The median IL-1*β* differences were −28.38 and −0.13 *μ*g/g in the NP and ET groups, respectively. A negative median value means that the cytokine decreased after scaling. The IL-1*β* difference was larger in the ET group than that in the NP group.

**Figure 2:**
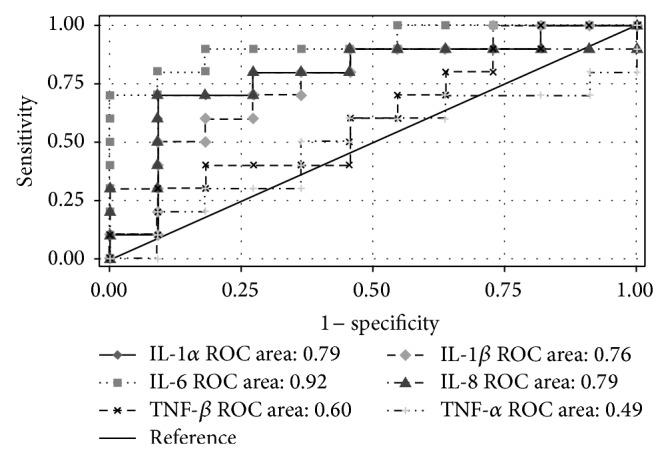
Receiver operating characteristic (ROC) curve and area under the curve (AUC) of proinflammatory cytokines for predicting the treatment outcome.

To evaluate the accuracy of predicting the periodontitis treatment outcome, ROC curves and AUC, based on the difference in proinflammatory cytokines, were calculated (Figure 2). The AUCs of IL-1*α*, IL-1*β*, IL-6, IL-8, TNF-*α*, and TNF-*β* were 0.79, 0.76, 0.92, 0.79, 0.49, and 0.60, respectively. The differences of IL-6 levels (cut point: 0.05 *μ*g/g) yielded a sensitivity and specificity of 90.0% and 81.82%, respectively, for predicting the periodontitis treatment outcome. Thus, the IL-6 difference is an effective marker for predicting the periodontitis treatment outcome.

## 4. Discussion

Inflammatory responses protect cells against periodontopathic bacteria [18, 19]. Bacterial DNA can activate inflammatory cytokine production [20]. Proinflammatory cytokines play a vital role in bone remodeling modulation [21]. High baseline cytokine levels could affect the effect of treatment. The baseline proinflammatory cytokines were not significantly different between the NP and ET groups in this study. Although differences in TNF-*α* and TNF-*β* were not correlated with the periodontal disease treatment outcome, differences in IL-1*α*, IL-1*β*, IL-6, and IL-8 were significantly higher in the ET group in this study. These results imply that scaling stimulated IL-1*α*, IL-6, and IL-8 and that IL-1*β* may be useful as a treatment progress biomarker of periodontitis.

Biologically active pro-IL-1*α* and pro-IL-1*β* are both synthesized in the cytoplasm and cleaved by other proteins to generate IL-1*α* and IL-1*β*, respectively [22]. Interleukin-1*α* located in the membrane acts as an intracellular transcriptional regulator; IL-1*β* regulates innate immunity and stimulates connective tissue turnover [23].* P. gingivalis* produced IL-1*β* in human periodontitis tissue and in a periodontitis activation animal model [24]. Salivary IL-1*β* is significantly higher in patients with severe periodontitis than in healthy controls; however, IL-1*β* does not differ between patients with mild periodontitis and healthy controls [9]. In this study, baseline salivary IL-1*β* increased significantly with the severity of periodontitis. The IL-1*β* difference was larger in the ET group than that in the NP group. For predicting periodontitis treatment outcomes, scaling-stimulated IL-1*β* is a superior biomarker than pretreatment salivary IL-1*β*.

Interleukin-6 has pleiotropic properties because it shares a common signaling pathway with the signal transducer glycoprotein 130 [25, 26]. For nonchallenged status, IL-6 was higher in gingival fibroblasts than in periodontal ligament fibroblasts; when challenged by* P. gingivalis*, the gingival fibroblasts of 4 of 6 subjects induced more IL-6 than did periodontal ligament fibroblasts [27]. Recent studies have shown that IL-6 is significantly higher in patients with chronic periodontitis than in healthy controls [8, 28]. Teles et al. showed that salivary IL-6 was lower in patients with chronic periodontitis than in healthy subjects; however, no statistical differences were found between the patients with chronic periodontitis and healthy subjects [29]. According to these reports, IL-6 may not be used as a biomarker for the diagnosis of periodontitis. In this study, the AUC of the difference in IL-6 after scaling was 0.90, suggesting that the difference in IL-6 is an effective biomarker for predicting the periodontitis treatment outcome.

Compared with the gingival fibroblast tissue from healthy subjects, that from periodontal disease patients produced higher IL-1 before and after* P. gingivalis* challenge and pretreating gingival fibroblasts with IL-1*α* enhanced IL-6 production [30]. Previous studies have shown significant correlations between IL-6 and IL-1*β* (*r* = 0.94) or IL-8 (*r* = 0.96) in gingival fibroblasts and periodontal ligament fibroblasts (*r* = 0.83 for IL-6 and IL-1*β*; *r* = 0.93 for IL-6 and IL-1*β*) [27]. In this study, significant correlations were also observed between salivary IL-6 and IL-1*β* (*r* = 0.72 and *P* < 0.01) of patients with periodontitis. Salivary IL-1*β* and IL-6 significantly increased with the severity of periodontitis. These two proinflammatory cytokines are sensitive to pathogen infection and can reflect the severity of periodontitis.

Studies have also revealed that an increase in the gene expression of IL-1*β*, IL-6, IL-8, and TNF-*α* occurs in response to a* P. gingivalis* challenge in primary human gingival fibroblasts and periodontal ligament fibroblasts, whereas the normal T cell expression and secretion are regulated. The responsiveness of fibroblasts from different donors is similar; this may be useful in determining the vulnerability to periodontitis [27]. An in vitro study showed that a* P. gingivalis* challenge can enhance IL-8 but not IL-6 production in human gingival fibroblasts, suggesting that IL-6 and IL-8 are produced via different pathways [4]. Scaling-stimulated IL-6 was significantly correlated with baseline IL-1*α*, IL-1*β*, IL-6, IL-8, and TNF-*α*. Interleukin-6 has proinflammatory properties and is involved in numerous acute inflammatory and pathologic processes, such as the promotion of bone resorption [22]. Moreover, IL-6 is emerging as a crucial mediator and a novel therapeutic target for chronic inflammatory diseases and cancer [7].

The limitation of this study is that saliva specimens after treatment could not be collected for all of the subjects; the specimens of only 15 subjects were collected after completion of the periodontal treatment. Hence, the collection of specimens after treatment was incomplete and those specimens had not been used to determine the proinflammatory cytokines because of financial reasons. In this study, the significance of the cytokine changes after treatment could not be investigated in depth. In addition, proinflammatory cytokines significantly increased with the severity of periodontitis, and stimulated IL-6 was found to be an effective marker for predicting the periodontitis treatment outcome. It would be useful to further investigate IL-6 as a novel therapeutic target for improving periodontitis treatment efficacy in future studies.

## Figures and Tables

**Table 1:** Demographic characteristics and conventional periodontitis risk factors of study subjects by treatment outcome strata.

	NP group (*N* = 12)	ET group (*N* = 10)	*P* value
	Median (Q1–Q3)		
Age, years	61.5 (46–71)	56.0 (44–60)	0.37^a^

Distribution of subjects	*N* (%)	*N* (%)	

Gender			0.63^b^
Male	5 (41.67)	4 (40.00)	
Female	7 (58.33)	6 (60.00)	
Education			0.41^b^
High school	4 (33.33)	2 (20.00)	
University or above	8 (66.67)	8 (80.00)	
Smoking			0.22^b^
Nonsmokers	10 (83.33)	6 (60.00)	
Smokers	2 (16.67)	4 (40.00)	
Alcohol consumption			0.71^b^
Never or occasional	11 (91.67)	9 (90.00)	
Regular	1 (8.33)	1 (10.00)	
Betel nut chewing			0.19^b^
Nonchewer	12 (100.00)	8 (80.00)	
Chewer	0 (0.00)	2 (20.00)	
Dental visiting pattern			0.96^b^
Regular visits (<1 year)	8 (66.37)	9 (90.00)	
Irregular visits (≥1 year)	4 (33.33)	1 (10.00)	
Tooth cleaning frequency			0.97^b^
<2 times/day	1 (8.33)	3 (30.00)	
≥2 times/day	11 (91.67)	7 (70.00)	

^a^Mann-Whitney *U* test.

^b^Fisher's exact test.

NP group: nonprogress treatment group.

ET group: effective treatment group.

**Table 2:** Baseline proinflammatory cytokine profiles of study subjects by treatment outcome strata.

	NP group (*N* = 12)	ET group (*N* = 10)	*P* value^a^
	Median (Q1–Q3)		
IL-1*α*	120.18 (12.58–167.16)	67.97 (52.3–190.12)	0.97
IL-1*β*	28.55 (3.15–119.49)	5.99 (0.18–14.8)	0.07
IL-6	5.21 (1–9.46)	1.24 (0.78–4.24)	0.15
IL-8	125.68 (50.05–184.49)	50.8 (16.9–138.74)	0.19
TNF-*α*	0.00 (0.00–0.11)	0.10 (0.00–0.12)	0.18
TNF-*β*	0.18 (0.16–0.21)	0.21 (0.17–0.23)	0.19

^a^Mann-Whitney *U* test.

**Table 3:** Correlation between baseline salivary proinflammatory cytokines in patients with periodontitis.

Baseline	Baseline					
	IL-1*α*	IL-1*β*	IL-6	IL-8	TNF-*α*	TNF-*β*
	Correlation coefficients (*P* value)					
IL-1*α*	1.00					

IL-1*β*	0.46^*^	1.00				
	(0.03)					

IL-6	0.26	0.72^**^	1.00			
	(0.26)	(0.0002)				

IL-8	0.32	0.60^**^	0.43	1.00		
	(0.16)	(0.0043)	(0.05)			

TNF-*α*	−0.14	−0.38	−0.33	0.00	1.00	
	(0.55)	(0.09)	(0.14)	(1.00)		

TNF-*β*	−0.42	−0.46^*^	−0.41	−0.07	0.56	1.00
	(0.06)	(0.04)	(0.07)	(0.77)	(0.01)	

^*^ *P* < 0.05.

^**^ *P* < 0.01.

**Table 4:** Correlation between baseline and stimulated salivary proinflammatory cytokines in patients with periodontitis.

Stimulated	Baseline					
	IL-1*α*	IL-1*β*	IL-6	IL-8	TNF-*α*	TNF-*β*
	Correlation coefficients (*P* value)					
IL-1*α*	0.66^**^	0.37	0.38	0.02	−0.4	−0.35
	(0.001)	(0.1)	(0.09)	(0.93)	(0.08)	(0.12)

IL-1*β*	0.42	0.44^*^	0.31	0.08	−0.36	−0.28
	(0.06)	(0.04)	(0.17)	(0.72)	(0.1)	(0.22)

IL-6	0.63^**^	0.47^*^	0.60^**^	0.26	−0.48^*^	−0.41
	(0.002)	(0.03)	(0.004)	(0.26)	(0.03)	(0.07)

IL-8	0.38	0.22	0.14	0.25	−0.30	−0.18
	(0.09)	(0.34)	(0.53)	(0.27)	(0.18)	(0.43)

TNF-*α*	0.29	0.25	0.03	−0.001	−0.26	−0.12
	(0.21)	(0.28)	(0.90)	(1.00)	(0.26)	(0.61)

TNF-*β*	−0.02	−0.24	−0.46	−0.14	0.27	0.32
	(0.92)	(0.29)	(0.03)	(0.54)	(0.24)	(0.16)

^*^ *P* < 0.05.

^**^ *P* < 0.01.

**Table 5:** Difference in proinflammatory cytokines between patients with and without clinical treatment progress.

Difference in proinflammatory cytokines	NP group (*N* = 12)		ET group (*N* = 10)		*P* value^a^
	Median	Q1–Q3	Median	Q1–Q3	
IL-1*α*	−11.97	−39.08–45.53	142.70	37.5–188.77	0.02
IL-1*β*	−28.38	−118.3–2.99	−0.13	−14.53–0.86	0.04
IL-6	−0.72	−7.19–0.05	3.33	2.76–10.18	0.0014
IL-8	−87.31	−133.23–−38.42	7.84	−51.79–64.65	0.02
TNF-*α*	0.11	0.00–1.02	0.41	−0.11–1.47	0.97
TNF-*β*	−0.01	−0.04–0.08	0.01	−0.02–0.16	0.45

^a^Mann-Whitney *U* test.

NP group: nonprogress treatment group.

ET group: effective treatment group.
